# Effect of Fibrillization pH on Gelation Viscoelasticity and Properties of Biofabricated Dense Collagen Matrices via Gel Aspiration-Ejection

**DOI:** 10.3390/ijms24043889

**Published:** 2023-02-15

**Authors:** Ehsan Rezabeigi, Gabriele Griffanti, Showan N. Nazhat

**Affiliations:** Department of Mining and Materials Engineering, McGill University, Montreal, QC H3A 0C5, Canada

**Keywords:** collagen, pH, gel aspiration-ejection (GAE), rheological properties, injection, densification

## Abstract

Reconstituted hydrogels based on the self-assembly of acid-solubilized collagen molecules have been extensively used as in vitro models and precursors in biofabrication processes. This study investigated the effect of fibrillization pH—ranging from 4 to 11—on real-time rheological property changes during the gelation of collagen hydrogels and its interplay with the properties of subsequently biofabricated dense collagen matrices generated via automated gel aspiration-ejection (GAE). A contactless, nondestructive technique was used to characterize the temporal progression in shear storage modulus (G’, or stiffness) during collagen gelation. There was a relative increase in G′ of the hydrogels from 36 to 900 Pa with an increase in gelation pH. Automated GAE, which simultaneously imparts collagen fibrillar compaction and alignment, was then applied to these precursor collagen hydrogels to biofabricate native extracellular matrix-like densified gels. In line with viscoelastic properties, only hydrogels fibrillized in the 6.5 < pH ≤ 10 range could be densified via GAE. There was an increase in both fibrillar density and alignment in the GAE-derived matrices with an increase in gelation pH. These factors, combined with a higher G′ in the alkaline precursor hydrogels, led to a significant increase in the micro-compressive modulus of GAE-densified gels of pH 9 and 10. Furthermore, NIH/3T3 fibroblast-seeded GAE-derived matrices densified from gels fibrillized in the pH range of 7 to 10 exhibited low cell mortality with >80% viability. It is anticipated that the results of this study can be potentially applicable to other hydrogel systems, as well as biofabrication techniques involving needles or nozzles, such as injection and bioprinting.

## 1. Introduction

Collagen gels have been extensively used in tissue engineering due to their outstanding biocompatibility and vital role as the main native extracellular matrix component. Type I collagen fibrils of diameters ranging from 20 to >100 nm [1,2,3,4], constitute ~30% of the total protein content in humans [5,6,7,8,9]. In vitro reconstitution of type I collagen gels relies on the self-assembly of acid-solubilized collagen molecules, by adjusting their pH and temperature close to those of physiological conditions, thus forming a fibrous mesh encasing an aqueous solution [10,11]. The resulting collagen fibrils are mostly stabilized by electrostatic and hydrophobic interactions between side chains of amino acids among adjacent fibrils [12]. It is well known that the in vitro self-assembly of collagen is affected by the ionic strength of the aqueous solution, as well as its temperature and pH, which influence the protein charge distribution and electrostatic and hydrophobic interactions [1,12,13,14,15,16,17,18].

Upon fibrillization, highly hydrated collagen (HHC) gels are typically formed with low collagen concentrations or fibrillar densities (i.e., <1 wt.%). While HHC gels are capable of incorporating cells and can be used as in vitro models [4,11,19,20,21,22], their poor structural and mechanical properties limit their tissue engineering applications [2,4,21,23,24]. Alternatively, densified collagen gels with enhanced mechanical properties can be generated via unconfined, uniaxial plastic compression [2,16,25], confined compression [26], biaxial compression [27], and gel aspiration-ejection (GAE) [24,28,29,30], among other techniques [31,32] (reviewed in [33]). In particular, GAE is a promising technique that can rapidly produce injectable scaffolds of various geometries and micro-architectures [24], which enable the delivery of both seeded cells [23,34,35,36] and incorporated bioactive materials [4]. Additionally, the ability of an automated-GAE approach to biofabricate tissue-mimicking 3D structures of cell-seeded densified collagen gel blocks with various properties, shapes, and sizes, has been demonstrated [24].

As most collagen densification processes are applied to pre-fabricated HHC gels, the viscoelastic properties of these precursor gels can be crucial during their densification. To this end, the ability to monitor the real-time rheological properties during gelation can provide critical information on the solid- and liquid-like behavior of HHC gels [9,11,19,22,37,38]. Moreover, fibrillization pH has been shown to be an easy-to-control and effective parameter that modulates the in vitro self-assembly of collagen [1,12,13,39]. For example, it has been shown that the kinetics of collagen fibril formation, as well as the stiffness and water uptake of HHC gels, can be significantly affected by the pH [12,20,38,39]. Therefore, in this study, the effect of fibrillization pH on real-time changes in stiffness during the collagen gel formation as well as the interplay between these rheological properties and the properties of GAE-densified matrices were investigated. It is anticipated that the results of this study can be potentially applicable to other hydrogel systems and biofabrication techniques involving needles or nozzles, such as injection and bioprinting.

## 2. Results and Discussion

### 2.1. Turbidity Measurements

**Figure 1:**
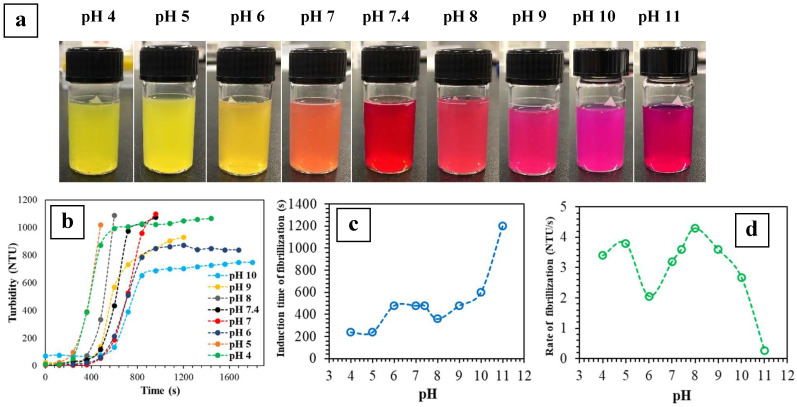
Turbidity measurement of collagen gelation: (**a**) Collagen solutions with various pH. Images were acquired immediately after the various collagen/MEM/NaOH solutions were prepared. (**b**) Change in turbidity of collagen solutions of various pH values as a function of time at 37 °C (note that the curve of pH 11 could not be presented here along with other curves due to its long gelation). The upper limit of the turbidimeter used here was 1100 NTU. (**c**) Induction time, and (**d**) rate of fibrillization as a function of pH generated from the corresponding turbidity-time graphs (*n* = 2).

Figure 1a–d presents the change in turbidity of collagen solutions with pH 4 to 10 over time at 37 °C. No change in turbidity was detected for systems of pH 3 and 12 (i.e., no gelation). All other systems exhibited an induction time of fibrillization (i.e., lag phase [19,39]), a period in which there was no change in turbidity. This was followed by a rapid increase in turbidity, which can be attributed to the advancement in fibrillization (Figure 1b). The final turbidity values after the rapid increase are arbitrary, especially since the solutions exhibited different colors at various pH (Figure 1a) that may also affect light transmission, which is a key factor in this analysis [11,19,39,40,41].

Figure 1c indicates that there was a delay in fibrillization with increasing pH, such that the system with pH 11 exhibited a long induction time of ~1200 s. This system also demonstrated the lowest rate of fibrillization (Figure 1d), thus prolonging the gelling process.

### 2.2. Viscoelastic Properties

**Figure 2:**
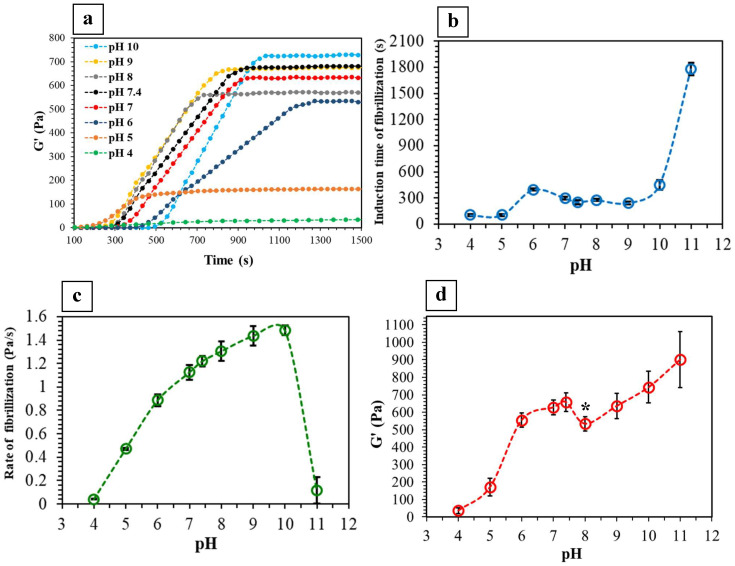
Real-time rheological measurement of collagen gelation: (**a**) Shear storage modulus (G′) of collagen solutions of various pH as a function of time at 37 °C (note that the curve of pH 11 could not be presented here along with other curves due to its long gelation). (**b**) Induction time, (**c**) rate of fibrillization, and (**d**) G′ of the HHC gels, as a function of pH at 37 °C (* *p* < 0.05; *n* = 5).

Figure 2a presents the change in G′ of collagen solutions with pH ranging from 4 to 10 as a function of time at 37 °C. G′ was used as the indication of solid-like behavior (stiffness) and gelation of the systems over time [9,11,19]. Gelation was initiated at different times, as reflected by the rapid increase in G′ values [9,19]. These values then stabilized, indicating the physical crosslinking of the fibrils and their entanglement within the gels [9,19,22]. In contrast, there was no change in G′ detected in either highly acidic (pH = 3) or alkaline (pH = 12) solutions, confirming no gelling, as also indicated by turbidity measurements.

Figure 2b presents the induction time of fibrillization as a function of collagen solution pH. Similar to the turbidity measurements (Figure 1c), there was a significant delay in the fibrillization of the pH 11 solution. On the other hand, the rate of fibrillization increased up to pH 10 and then significantly decreased at pH 11 (Figure 2c). It has been discussed that the rate of fibrillization is mainly controlled by electrostatic interactions, which are affected by pH as a result of altering the net electrostatic charge of the fibrils [8,39,42].

The induction time of fibrillization in Figure 2b exhibited a similar trend with a slight shift in time compared to that obtained from the turbidity measurements (Figure 1c and Figure S1a). This indicates that the rapid increase in G′ of the solutions corresponded to their sudden increase in turbidity, although the G′ measurements detected the changes more precisely. On the other hand, the trends in the rate of fibrillization obtained from these two methods (Figure 1d and Figure 2c) are different (Figure S1b), highlighting the fact that the changes in G′ of the solutions did not necessarily correspond to their turbidity progression. In other words, there is no guarantee that the change in G′ of the solutions during fibrillization occurs exactly at the same rate as the change in their turbidity.

As shown in Figure 2d, there was an increase in HHC stiffness with an increase in fibrillization pH. This can be attributed to greater physical crosslinking and fibrillar entanglement within the gels. Similarly, Raub et al. [20] demonstrated a similar increase in G′ of collagen gels by increasing the pH of fibrillization from 5.5 to 9. Moreover, Yamamura et al. [38] reported that by increasing the pH in the range of 5 to 8, the relaxation modulus of collagen gels linearly increased (i.e., gels became stiffer) and then plateaued.

The isoelectric point (IEP) of collagen solution with physiological ionic concentration can be approximated at pH 7.8 [43,44,45,46]. By changing the pH of the collagen solution from its IEP, surface charges appear on the fibrils, thereby forming electrostatic interactions [43,44]. A molecule of type I collagen at pH 7 contains 232 negatively and 258 positively charged sites that interact with those of the neighboring molecules during fibrillization [15], whereas, in a more alkaline solution, the number of negative charges increases [15,21]. The net electrostatic charges at a slightly alkaline pH of ~8 (i.e., close to IEP) in this study may have approached a balance [47,48] creating a local reduction in G′ of this system (Figure 2d). On the other hand, for gels of pH ≥ 9, the formation of helical structures may be promoted, resulting in higher G′ values [9,49]. In addition, higher pH favors the formation of longer, more rigid, and tightly packed fibrils, all of which contribute to higher G′ values [9,12,49]. Furthermore, the intermolecular forces in highly alkaline systems may prevent the deformation of more flexible regions within the fibrillar network, thereby resulting in the strain hardening of gels with higher pH [12,14,15,50]. 

### 2.3. GAE-Densified Collagen Gels

Automated GAE was applied on HHC gels fibrillized in the range 6.5 < pH ≤ 10 to produce densified collagen matrices. Sections S2 and S3 (Supplementary Material) provide further information on the GAE process (Figure S2) and structural analysis through infrared spectroscopy (Figure S3) [14,37,51,52,53].

#### 2.3.1. Fibril Alignment and CFD

Collagen fibrils exhibit different levels of alignment in various native tissues such as blood vessels, tendons, bone, and skin [4,7,23,24,54]. Although fibrillar alignment can be an important requirement in designing collagen-based tissue-mimicking matrices, it is largely overlooked in biofabrication techniques. GAE is a densification method that allows for a shear-induced fibril alignment during the aspiration process which can be tailored by controlling processing parameters, such as needle size and contact surface roughness [4,23,24,29,55].

**Figure 3:**
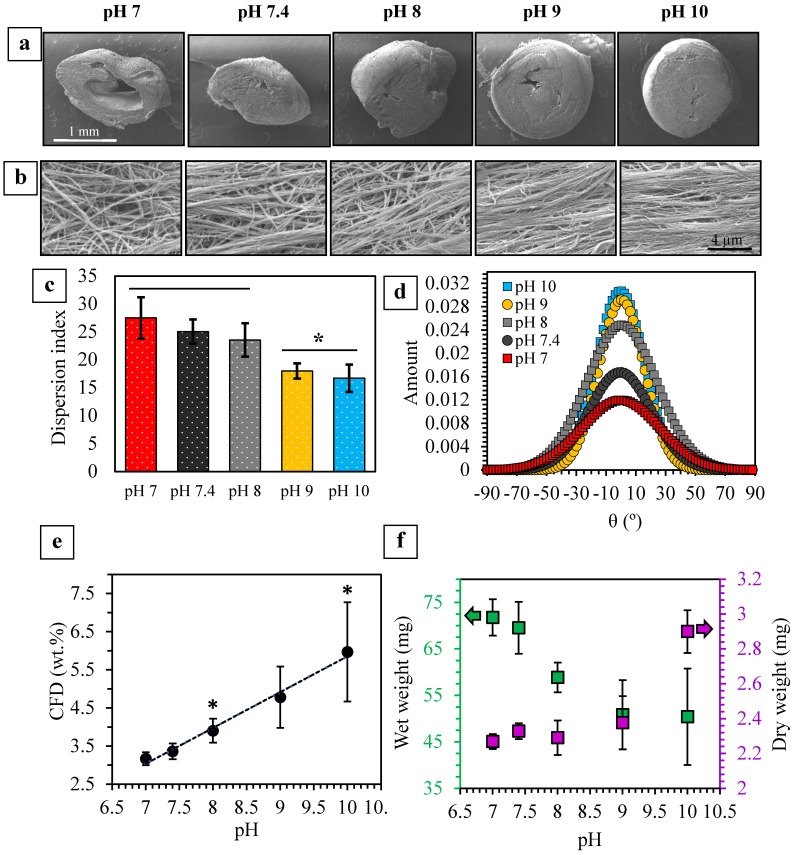
pH-regulated properties of the GAE-densified collagen gels: (**a**) Low magnification cross-sectional and (**b**) higher magnification SEM images of the surface of the GAE-densified gels. (**c**) Collagen fibril directionality (* *p* < 0.05; *n* = 10) and (**d**) mean collagen fibril dispersion angles of the gels (*n* = 10). There was an increase in fibrillar alignment (lower dispersion indices) with an increase in fibrillization pH. (**e**) CFD (* *p* < 0.05; *n* = 7) and (**f**) dry and wet weights of the GAE-densified matrices gels as a function of fibrillization pH.

Figure 3a–d presents the SEM images of the GAE-densified collagen gels fibrillized at various pH as well as their levels of fibrillar alignment. Gels fibrillized at a higher pH (e.g., 9 and 10) appeared to generate more compact and cylindrical GAE-densified matrices (Figure 3a) when compared to those gelled at lower pH (e.g., pH 7). Higher degrees of physical crosslinking and fibrillar entanglements within gels of higher pH may be key in maintaining their original cylindrically shaped hydrated gels. In other words, stiffer gels led to the formation of denser samples which undergo less shrinkage during drying. Moreover, an increase in pH appeared to favor greater fibrillar alignment (Figure 3b–d). Since thinner and longer fibrils are more likely to form at a higher pH (e.g., pH 9 and 10) [10,20], they can better remodel as an integrated network during aspiration, leading to higher degrees of alignment. These observations suggest that in addition to the previously established parameters [24], the fibrillization pH of collagen gels may also influence their fibril alignment.

It has previously been shown that in densified collagen gels, CFD and hydraulic permeability are inversely related [24,31]. Moreover, an increase in CFD leads to increased stiffness and may improve cellular function by stimulating intracellular signaling cascades [24,56]. Figure 3e quantitatively shows that the gels became relatively denser as the pH increased from 7 to 10. This increase in CFD is mainly related to the change in the wet weights (Figure 3f) of the gels since there is no significant change in their dry weights (except for the pH 10 gel where its high dry weight essentially contributed to its higher CFD). In other words, the decreasing trend in wet weights of the gels indicates that the as-ejected densified gels of higher pH are less hydrated, which further confirms their denser network. These results are also consistent with previous studies which showed that in HHC gels, the average pore size and area fraction decreased for systems of higher pH [38,49].

#### 2.3.2. Mechanical Properties 

**Figure 4:**
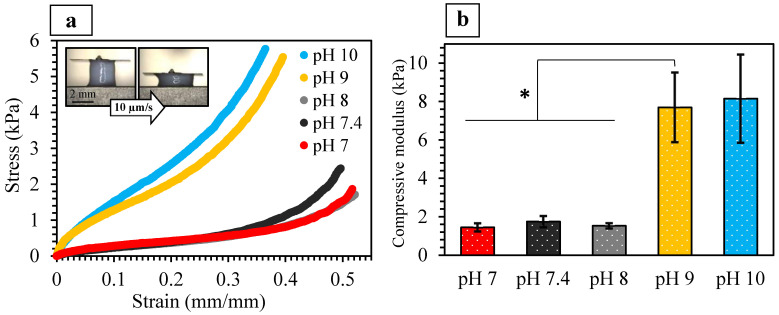
Mechanical properties of GAE-densified collagen gels: (**a**) Micro-compression stress-strain curves, and (**b**) compressive modulus of GAE-densified collagen gels of various pH (* *p* < 0.05; *n* = 7). Specimens were positioned such that their aspiration direction was parallel to the direction of the applied force.

The stiffness of densified collagen matrices has been shown to be dependent on their CFD, fibrillar alignment, diameter, length, and stiffness as well as degree of physical crosslinking [1,8,9,22,23,38,49]. Figure 4a presents representative micro-compressive stress-strain curves of the GAE-densified gels of various pH. There was a five-fold increase in the stiffness when the gels were fibrillized at pH 9 and 10 (Figure 4b), which can be attributed to their simultaneously increased CFD, fibril alignment, and G′ values. In contrast, lower levels of CFD (Figure 3e) and fibril alignment (Figure 3b–d) in gels of pH 7 and 7.4 contributed to their lower compressive modulus (1.45 ± 0.21 and 1.75 ± 0.29 kPa, respectively). Interestingly, the compressive modulus of the pH 8 gels is similarly low (1.53 ± 0.14 kPa), although its CFD and fibrillar alignment were higher than those of pH 7 and 7.4 gels. This may be attributed to the lower G′ of the precursor HHC gel of pH 8 (Figure 2d). Figure S4 presents the correlation between the compressive modulus of the densified gels and all these parameters as a function of pH.

By using a confined compression setting, Achilli et al. [49] demonstrated that with an increase in pH from 7.5 to 10, the mechanical responses of collagen-based gels were increased. On the other hand, Marelli et al. [17] did not observe any significant difference in the tensile apparent modulus in plastically compressed and rolled dense collagen gels of pH 7.4, 8.2, and 9. However, distinct from GAE, such plastically compressed gels do not exhibit fibrillar alignment.

It has been shown that when formed under acidic conditions, collagen fibrils are normally thicker than those when formed under either neutral or alkaline conditions [1,10,20,38], although some studies have stated otherwise [13]. It was also previously suggested that the fibril diameter may only affect the mechanical properties at small strains [1,20,57]. Nevertheless, the length of the fibrils may have a greater effect on the viscoelastic and mechanical properties of the gels as compared to their thickness. Thus, it can be postulated that the higher fibril aspect ratios at a higher pH [10,20] may have contributed to the enhanced mechanical properties of the gels when fibrillized at pH 9 and 10. 

#### 2.3.3. Seeded Fibroblast Viability and Mortality

**Figure 5:**
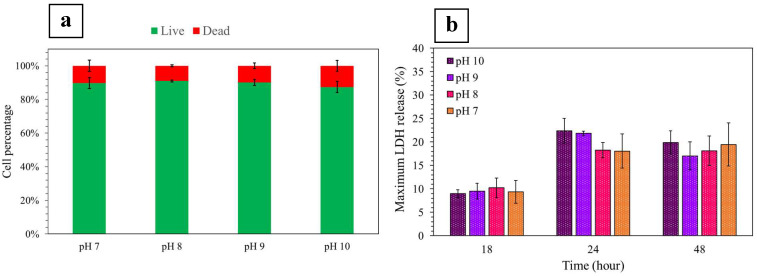
Cell viability assessment of NIH/3T3 fibroblast-seeded GAE-densified collagen gels of various pH: (**a**) Viability and mortality percentages, and (**b**) release of LDH from seeded cells.

Cell viability assessment of various systems post densification, bioprinting, or injection is an essential characterization step in the biofabrication process [24,58]. In the current study, in order to investigate cellular responses to various environments characterized by different pH, NIH/3T3 fibroblasts were seeded in HHC gels at pH 7, 8, 9, and 10, which were then densified through GAE. Cells were extracted from the gels after 48 h and as shown in Figure 5a, the percentages of viable and dead cells were similar in all systems, demonstrating >80% viability. Although the mean value of viable cells appeared slightly lower at pH 10, this difference was not significant (*p* > 0.05) suggesting that cell viability was not impacted when gels were cast in precursors of different pH followed by densification via GAE. These results are consistent with those generated from cellular LDH release when seeded in gels at pH 7, 8, 9, and 10 (Figure 5b). No difference between the maximum LDH release of cells seeded in the systems of various pH was observed up to 48 h, thus further confirming that the pH of the HHC precursors did not affect cell survival. 

In line with these results, it has previously been shown that the viability of NIH/3T3 fibroblasts was not affected when seeded in gels fibrillized at pH values of 7.4, 8.2, and 9, and subsequently densified via plastic compression [59]. These results also appeared to be consistent with another study on cell viability in dense collagen gels with a pH of 7.4 and 8.5 [60]. Indeed, it was demonstrated that the slightly alkaline pH of 8.5 of the HHC precursors did not affect the cell survival rate in plastically compressed collagen gels [60]. For the wider pH range reported in this study, the hypothesis is that cell exposure to the alkaline pH is limited to the short gelation and GAE processing timeframe and did not induce cell damage. Furthermore, since the cell-seeded GAE-derived dense collagen gels were cultured in medium of neutral pH, the neutrality of the pH within the gel micro-environment was rapidly reestablished.

## 3. Materials and Methods

### 3.1. Assessment of Gelation by Turbidity and Rheological Analyses

To prepare the collagen solutions, 3 mL of 10×-concentrated Modified Eagle Medium (10× MEM; First Link Ltd., Birmingham, UK) was mixed with up to 120 µm of 5 M NaOH. Next, 12 mL of rat-tail tendon type I collagen (2.05 mg/mL, in 0.6% acetic acid, First Link Ltd., Birmingham, UK) was slowly added into the solution under gentle mixing. At this point, the pH (measured using a Thermo Scientific Orion Star A211 pH-meter) of the solutions was adjusted to the target values of 3, 4, 5, 5.5, 6, 6.5, 7, 7.4, 8, 9, 10, 10.5, 11, and 12 via dropwise addition of 5 M NaOH. During the entire process, the containers were kept on ice to prevent gelation. The gelation process of all the resulting collagen/MEM solutions was monitored using a turbidimeter (TB300 IR; 0.01 < NTU < 1100). Here, aliquots of 11 mL were used to take the measurements every 2 min at 37 °C, and the change in turbidity of the solutions was plotted as a function of time (*n* = 2). 

The changes in shear storage (elastic) modulus (G′) versus times of the same collagen/MEM solutions were also analyzed using an ElastoSens^TM^ Bio^2^ (Rheolution Inc., Canada). This device accurately measures the real-time viscoelastic properties of soft biomaterials under controlled conditions via a nondestructive technique using gentle mechanical vibrations [61,62,63]. Aliquots of 7 mL of the solutions were loaded into the instrument’s sample holders and tested in both soft (*n* = 5) and stiff (*n* = 5) modes at 37 °C for 30–45 min and up to 4 h. G′ was recorded as a function of time using a 60 s resolution. Section S4 (Supplementary Material) gives further information on the G′-time curves. 

In both turbidity and stiffness measurements, the induction time of fibrillization (i.e., the onset of gelation) was considered as the time at which there was a rapid increase in either turbidity or G′, respectively. The rate of fibrillization was calculated from the slope of the linear regression fitted (R^2^ ≥ 0.990 ± 0.002) to the portion in which there was a rapid increase in either turbidity or G′. Moreover, based on the G′ measurements, the final G′ of the HHC gels as a function of gelling pH was calculated by averaging the stabilized values in the plateau region of the corresponding G′-time curves.

### 3.2. Production of the Dense Collagen Gels via GAE

Automated GAE was applied on precursor HHC gels fibrillized under pH 7, 7.4, 8, 9, and 10 to generate dense collagen gel matrices (Figure S1). Aliquots of 1.5 mL solutions were cast into 48 well plates and placed in an incubator for ~30–40 min at 37 °C to gel, followed by densification via an automated-GAE device [24] coupled with a 12 G (inner diameter = 2.16 mm [4]) blunt-end stainless steel needle (McMaster-Carr, Cleveland, OH, USA) using the aspiration and ejection rate of 0.25 and 2 µL/s, respectively. The densified gels were then ejected into phosphate-buffered saline (PBS). The GAE system was rinsed once with deionized water (DIW) between sample productions. Some of the as-ejected samples were weighed before drying to calculate their collagen fibrillar density (CFD) as described below. 

### 3.3. Scanning Electron Microscopy (SEM)

GAE-densified gels were morphologically characterized through SEM. As-ejected dense collagen gels were dried through a stepwise solvent exchange in DIW/ethanol solutions (50/50, 40/60, …, 10/90, and 5/95 *v/v*), with 15 min immersion time in each medium, followed by immersing in 100% ethanol for 45 min and then in 1,1,1,3,3,3-hexamethyldisilazane (Sigma-Aldrich, Oakville, ON, Canada) for 30 min. The resultant samples were eventually dried overnight under ambient conditions (inside a fume hood). The morphological examination was carried out using a field-emission SEM (Quanta 450 FEG; FEI Corporation, Hillsboro, OR, USA) at 5 kV after coating with Pt using a sputter coater (Leica Microsystems EM ACE600). SEM images with 30,000× magnification were used to quantify the fibril alignment within each sample (*n* = 10) using the ImageJ (NIH, USA) software with an open source plug-in.

### 3.4. Collagen Fibrillar Density (CFD)

As-ejected dense collagen gels were weighed (wet weights; *Ww*) after gentle rolling on a glass slide to remove the excess surface fluid. Next, the samples were dried as described above and weighed again (dry weight; *Wd*). The CFD of the specimens (*n* = 7) was calculated using the (*Wd*/*Ww*) × 100 equation [24,29,56].

### 3.5. Micro-Compression Testing

As-ejected dense collagen gels were gently cut using a surgical blade to create shorter cylindrical specimens, which were kept hydrated in PBS before mechanical testing. Tests were carried out under unconfined, quasi-static micro-compression (Microsquisher, CellScale Biomaterials, Waterloo, ON, Canada) with a displacement rate of 10 µm/s. Specimens (*n* = 7) were placed in the instrument such that their aspiration direction was parallel to the direction of the applied force. Engineering stress-strain curves were then produced using the “force versus displacement” data given by the instrument and the initial cross-sectional areas and heights of the specimens. Slopes of the linear portion of these stress-strain curves were used to calculate the compressive modulus.

### 3.6. Seeded Cell Viability and Mortality

#### 3.6.1. Production of Fibroblast-Seeded Dense Collagen Gels

Passage 13 murine-derived NIH/3T3 (ATCC^®^ CRL-1658TM) fibroblasts were incubated at 5% CO_2_ and grown to 80% confluency at 37 °C. The expansion and growth medium consisted of Dulbecco’s Modified Eagle Medium (DMEM, Fisher Scientific, Ottawa, ON, Canada) supplemented with 10% bovine calf serum (HyClone Laboratories Inc., Logan, UT, USA) and 1% Penicillin Streptomycin antibiotic (Fisher Scientific, Ottawa, ON, Canada). Cells were seeded at a density of 2 × 10^5^ cells/mL, in collagen solutions which were gelled at pH 7, 8, 9, and 10. Gelling was enabled in an incubator with 5% CO_2_ atmosphere at 37 °C. As cast, cell-seeded HHC gels were then processed through automated GAE, as described above.

#### 3.6.2. Fibroblast Extraction from Cell-Seeded Gels

To investigate the effect of the gelation pH on the viability and mortality of seeded NIH/3T3 fibroblasts, cells were extracted from gels after 18, 24, and 24 h in culture. Gels were digested in DMEM supplemented with 100 units/mL collagenase type I (300 units/mg, Worthington Biochemical Corporation, Lakewood, NJ, USA) at 37 °C for 2 h. Extracted cells were pelleted through centrifugation. An aliquot was stained with Trypan Blue solution (Gibco, Grand Island, NJ, USA) which allowed for the determination of percentage cell viability/mortality using a hemocytometer.

#### 3.6.3. Detection of Cellular Lactate Dehydrogenase Release (LDH)

The effect of gelation pH on the mortality of seeded NIH/3T3 fibroblasts was investigated by monitoring cellular LDH release (LDH Cytotoxicity Detection Kit; Clontech, Mountain View, CA, USA) from cells cultured in the GAE-densified gels. Cell-free culture supernatant (100 µL aliquots) was collected in triplicate from triplicate samples up to 48 h after the application of GAE and then incubated with the reaction mixture according to manufacturer instructions. LDH activity was determined by measuring the absorbance of the samples at 490 nm using a Synergy H1 microplate reader (BioTek, Ottawa, ON, Canada) and then compared to the total LDH release by cells killed in DMEM containing 1% Triton X-100 (Promega Corporation, Madison, WI, USA). Results are expressed relative to maximum LDH release.

### 3.7. Statistical Analysis

The statistical significance of the measured values was determined using Student’s *t*-test in Excel at a significance level of *p* < 0.05.

## 4. Conclusions

In this study, it was shown that the G′ of HHC gels was highly dependent on their pH, demonstrating an increase from 36 to 900 Pa with an increase in pH from 4 to 11. On the other hand, a local minimum G′ at pH ~8 may be correlated with the isoelectric point of the solution. The effect of pH on the precursor HHC gels and their subsequent densification was further investigated using automated GAE, where densified collagen constructs were successfully produced via GAE from HHC gels of 6.5 < pH ≤ 10. By increasing the pH of the HHC gels from 7 to 10, the CFD of the densified gels increased from 3.2 to 6.0 wt.%, along with their fibrillar alignment and compressive modulus (1.45 to 8.15 kPa). Moreover, NIH/3T3 fibroblast-seeded GAE-derived dense collagen gels of pH 7 to 10 exhibited low levels of cell mortality with >80% viability. The results of this study can be applied to other hydrogel systems, densification methods, and biofabrication techniques involving either injection or extrusion.

## Data Availability

Not applicable.

## Supplementary Materials

The following supporting information can be downloaded at https://www.mdpi.com/article/10.3390/ijms24043889/s1.
