# Supplementary material for: Effect of Fibrillization pH on Gelation Viscoelasticity and Properties of Biofabricated Dense Collagen Matrices via Gel Aspiration-Ejection
Source: Int J Mol Sci. 2023 Feb 15;24(4):3889. doi: 10.3390/ijms24043889 (PMC9967780; doi:10.3390/ijms24043889)
Supplement: Supplementary file 1 [file ijms-24-03889-s001.zip › ijms-2129552-supplementary.pdf]

Supplementary Material

Effect of fibrillization pH on gelation viscoelasticity and properties of biofabricated dense collagen matrices via gel aspiration-ejection

Ehsan Rezabeigi, Gabriele Griffanti and Showan N. Nazhat *

Department of Mining and Materials Engineering, McGill University, Montreal, QC H3A 0C5, Canada
* Correspondence: showan.nazhat@mcgill.ca; Tel.: +1-(514)-398-5524

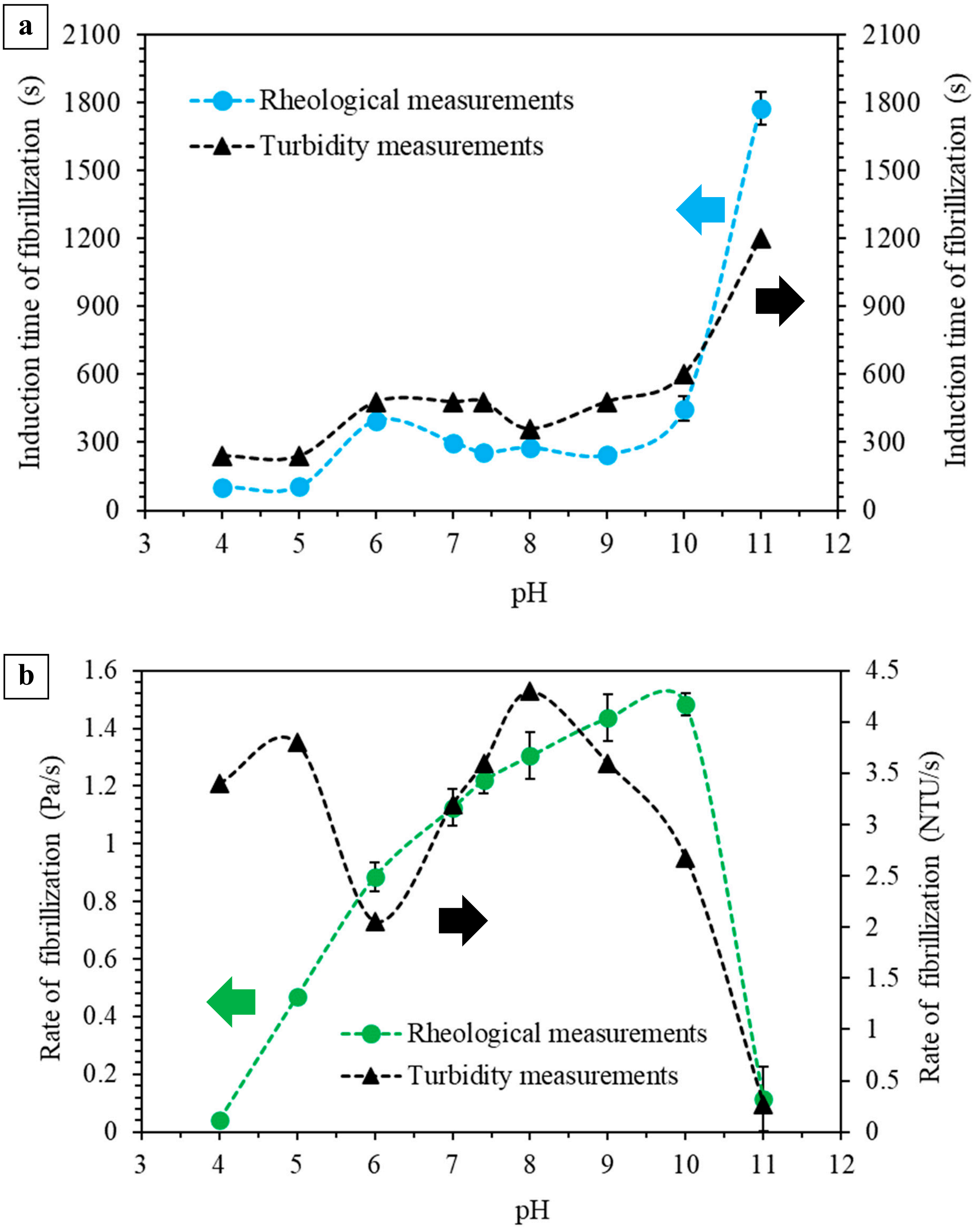

Figure S1. (a) Induction times and (b) rates of fibrillization obtained from the rheological and turbidity measurements and plotted for comparison. Figures 1c and 2b as well as Figures 1d and 2c were used to generate these plots a and b, respectively.

S2. Densification of HHC gels via GAE

Aspiration of HHC gels with pH 5, 5.5 and 6 was not feasible as the gel networks were not strong enough to withstand as a whole under the negative pressure (suction) leading to the rupturing of the gels. For example, the aspiration of the gel of pH 6, was either not successful (the gel could not be entirely aspirated); or the aspirated gels were fragile, breaking down during ejection. The aspiration of HHC gel of pH 6.5 was possible, however, the as-ejected gels were still fragile and hard to handle. Similarly, gels with high pH of 10.5 and 11 could not be aspirated. At such high pH, gels behave more solid-like (*i.e.*, with higher G'; Figure 2d) making it more difficult for the gel networks to yield and flow inside the needle. That is, for gels of higher pH which are stiffer, unbinding of the entanglements becomes difficult , which consequently interferes with their aspiration-ejection process.

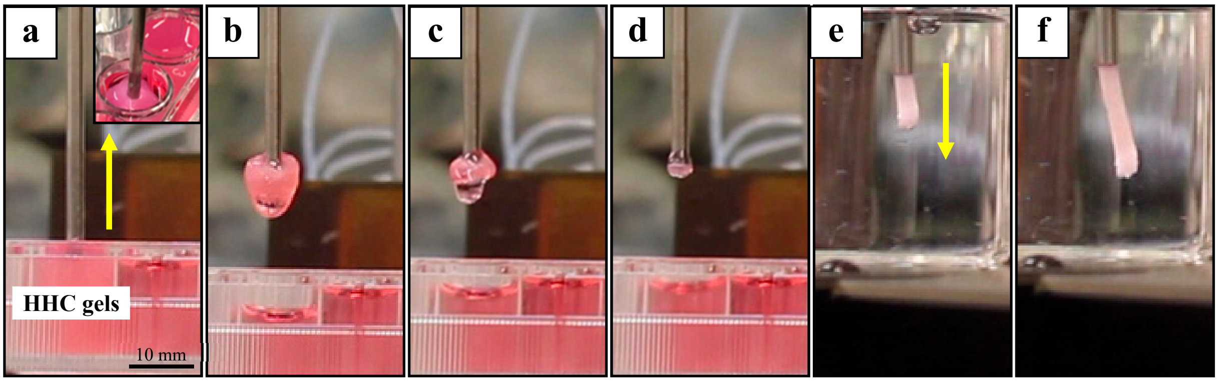

Figure S2. (a-d) Aspiration and (e-f) ejection steps in the GAE densification technique. Precursor HHC gel is aspirated into the needle while a part of the liquid is expelled out (b to c). The densified gel is then ejected into phosphate-buffered saline (PBS) (e and f).

S3. Attenuated total reflectance Fourier-transform infrared (ATR-FTIR) spectroscopy

Dried GAE-derived dense collagen matrix samples were examined using ATR-FTIR (Thermo Scientific Nicolet iS10). The spectra were collected between 4000 and 500 cm^{-1} with a resolution of 2 cm^{-1} and 64 scans per measurement. The spectra were normalized to the amide I peak (1635 cm^{-1}).

The spectra presented in Figure S3 indicate that all samples demonstrated similar patterns composed of the typical absorption bands of type I collagen, *i.e.,* amide I (C=O stretching) at 1635 cm^{-1}, amide II (N–H deformation and C–N stretching) at 1550 cm^{-1}, and amide III (N–H deformation and C–N stretching) at 1228 cm^{-1} as well as amide A (N–H and O–H stretching) at ~3305 cm^{-1} and amide B (C–H stretching) at 2930 cm^{-1}. All these main characteristics bands

appeared in the same positions indicating that the fibrillization pH range of 7 to 10 did not affect the structure of collagen. However, the unusual spike in the spectrum of sample of pH 10 in the range of 1800-4000 cm^{-1}, which may be caused by intense infrared light scattering, can be partly attributed to the increase in the number of hydroxyl groups forming hydrogen bridges within the gel network potentially promoting physical crosslinking [1-5].

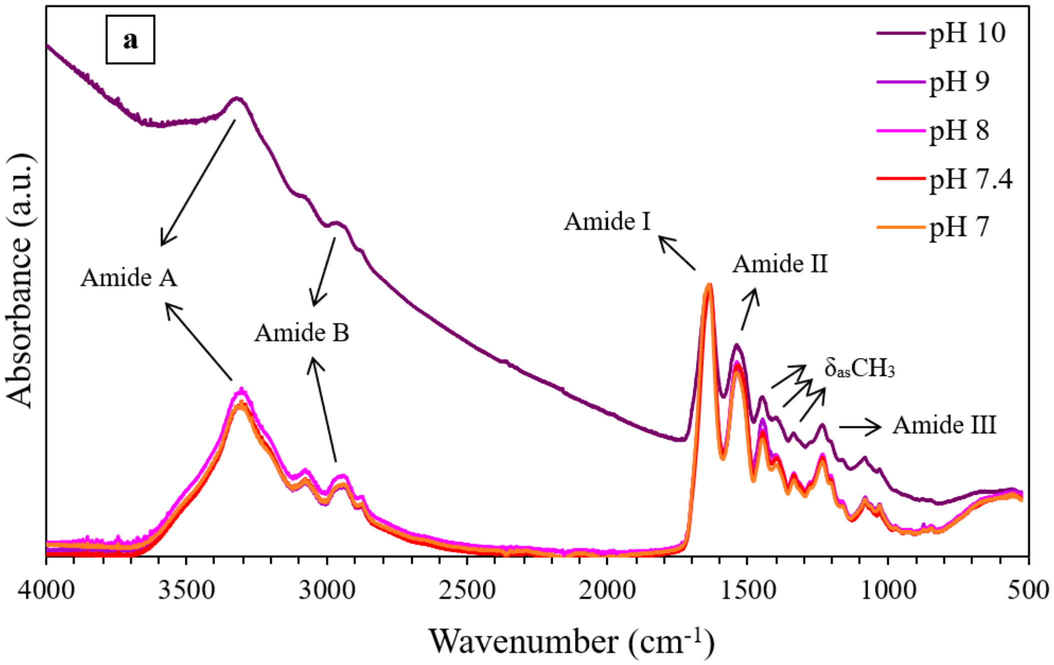

Figure S3. ATR-FTIR spectra of the GAE-derived densified gels produced from HHC gels of various pH. Spectra were normalized to the amide I band.

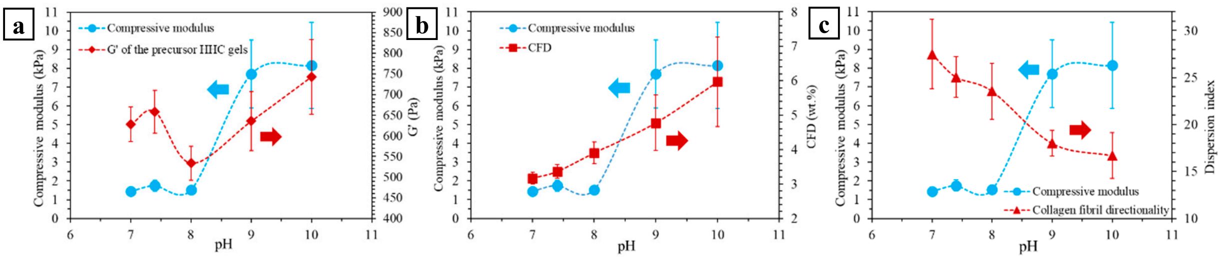

Figure S4. Correlation between the compressive modulus (Figure 4b) of the densified gels and (a) G' of the precursor HHC gels (Figure 2d); (b) CFD of the densified gels (Figure 3e); and (c)

collagen fibril directionality of the densified gels (Figure 3c), all as a function of pH. Note that the dispersion index (in graph c) is inversely related to the level of fibril alignment, *i.e.*, systems of lower pH having higher dispersion indices, exhibited lower levels of fibril alignment (Figure 3 b and c).

S4. Plotting the G'-time curves

Both the soft and stiff modes of ElastoSens^{TM} Bio^2 were used to accurately measure gels with G' values lower and higher than 500 Pa, respectively. The final G'-time plots were then constructed by combining the data of both modes to create the most accurate patterns (n = 5) for the solutions of various pH from loading until gelation. The only exceptions were the solutions of pH 4 and 5 which were solely tested in the soft mode since their G' values were below 500 Pa.

References

[1] Achilli *et al.* On the effects of UV-C and pH on the mechanical behavior, molecular conformation and cell viability of collagen-based scaffold for vascular tissue engineering. *Macromolecular Bioscience* **2010** *10* 307-316.

[2] Lian *et al*. ZnO/PVP nanoparticles induce gelation in type I collagen. *European Polymer Journal* **2016** *75* 399-405.

[3] Chang and Tanaka. FT-IR study for hydroxyapatite/collagen nanocomposite cross-linked by glutaraldehyde. *Biomaterials* **2002** *23* 4811-4818.

[4] Santos Silva Júnior et al. Effect of papain-based gel on type I collagen-spectroscopy applied for microstructural analysis. *Scientific Reports* **2015** *5* 11448.

[5] Vidal and Mello. Collagen type I amide I band infrared spectroscopy. *Micron* **2011** *42* 283-289.
